# Minimally Invasive Bipolar Technique for Scoliosis in Rett Syndrome—Results and Complications in a Series of 22 Cases

**DOI:** 10.3390/jcm14030849

**Published:** 2025-01-27

**Authors:** Alice Del Sal, Edouard Haumont, Manon Pigeolet, Mathilde Gaume, Guillaume Riouallon, Nadia Bahi Buisson, Agnes Linglart, Isabelle Desguerre, Stephanie Pannier, Lotfi Miladi

**Affiliations:** 1 Department of Pediatric Orthopedic Surgery, Hôpital Universitaire Necker—Enfants Malades, Paris Cité University, 75015 Paris, France; edouardhaumont@gmail.com (E.H.); manon.pigeolet@outlook.com (M.P.); mathilde.gaume@aphp.fr (M.G.); nadia.bahi-buisson@aphp.fr (N.B.B.); isabelle.desguerre@aphp.fr (I.D.); stephanie.pannier@aphp.fr (S.P.); 2 Faculty of Medicine, Université Libre de Bruxelles, 1050 Brussels, Belgium; 3 Department of Global Health and Social Medicine, Harvard Medical School, Boston, MA 02115, USA; 4 Department of Orthopedic Surgery, Hôpital Saint Joseph, 75014 Paris, France; griouallon@ghpsj.fr; 5 Department of Pediatric Endocrinology, Hôpital Universitaire Kremlin Bicetre, Paris Saclay University, 94270 Le Kremlin Bicêtre, France; agnes.linglart@aphp.fr

**Keywords:** scoliosis in Rett syndrome, bipolar technique, minimally invasive fusionless surgery, growth guided surgical technique, self-expanding rod, neuromuscular scoliosis

## Abstract

**Background**: This is a retrospective study. The aim of this study is to report the results of bipolar minimally invasive fusionless surgery for scoliosis in Rett syndrome with a minimum follow-up of 2 years. Conservative treatment is often not effective in Rett syndrome scoliosis. Posterior spinal fusion (PSF) has a high rate of complications; early surgery using traditional growing rods (TGRs) controls the deformity while preserving spinal and thoracic growth before arthrodesis. The need for surgical rod lengthening still has a high rate of complications and costs. **Methods**: We recorded the clinical and radiological outcomes of 22 consecutive patients with Rett scoliosis who underwent bipolar fusionless surgery with a mean follow-up of 56 months (24–99). We performed a bilateral construct with rods (with or without a self-sliding device) anchored proximally with four hook claws distally to the pelvis by ilio-sacral (IS) screws through a minimally invasive approach. **Results**: The Cobb angle was reduced from 74.4° initially to 28.9° postoperatively and to 25.7° at the last follow-up, which corresponds to a 65% correction of the initial deformity. The gain was maintained at the last follow-up. None of the patients required spinal fusion at skeletal maturity (55% of our patients reached skeletal maturity). There was a gain in body weight (27.97 kg at preoperative time and 33.04 kg at postoperative time). The surgical complication rate was 32%. **Conclusions**: We recorded the stable correction of deformities and weight gain over time using the bipolar minimally invasive fusionless technique with a reduced rate of complication compared to arthrodesis. The arthrodesis was not necessary at skeletal maturity, thanks to the delayed natural ankylosis of a fixed spine.

## 1. Introduction

Rett syndrome (RS) is an X-linked neurodevelopmental disorder that affects approximately 1 in 10,000 female births, primarily caused by mutations in the Methyl-CpG-binding protein 2 (MECP2) gene [1,2].

Mutations in the MECP2 gene on the X-chromosome account for the most common form of Rett syndrome (90–95%) [3]. Survival requires at least one functional MECP2-gene, making the mutation lethal in male embryos either in utero [4] or before two years of age [5].

RS is characterized by the loss of spoken language and hand function, along with the development of distinct hand stereotypies, first described in the 1960s by Andreas Rett. In a seminal paper, Bengt Hagberg and colleagues detailed the specific clinical features of the disorder and introduced the eponym we use today [6,7]. Clinical diagnosis is based on consensus criteria [8].

In 2006, RettSearch, an international network of Rett syndrome researchers, established updated diagnostic criteria. Following the regression phase, many individuals enter a period of stabilization, with some showing a partial recovery of lost skills.

Supporting clinical signs that aid in the diagnosis, RS includes decelerated head growth, breathing abnormalities, and the characteristic “Rett Gaze”, which is used for communication [9].

Patients with Rett syndrome also experience early and severe bone fragility, leading to an increased risk of fractures [10].

Scoliosis is the most common orthopedic complication in RS, occurring in 36–100% of cases [11,12,13,14], and is typically associated with MECP2 mutations [15]. Most patients develop a long C-shaped [16] thoracolumbar curve, with onset between the ages of 8 and 12 years [17]. Occasionally, kyphosis is also present. Initial treatment often involves conservative methods, such as bracing, though these rarely stop progression [18] more than 50% of patients will eventually require surgical correction to prevent severe respiratory and digestive disorders [17].

Historically, early PSF and the later use of TGR have been the primary treatments [19,20]. However, PSF does not allow normal lung development, and in some cases, this leads to the crankshaft phenomenon. TGR, while effective, often requires multiple revision surgeries due to complications such as deep surgical site infections, material failure, and bleeding. [21,22].

The minimally invasive bipolar technique [23], which includes a one-way self-expanding rod device [24,25], has been developed as an alternative for managing neuromuscular scoliosis. This technique minimizes the need for revision surgeries, reducing complications like blood loss, repeated anesthesia, and the risk of infection.

Given that this technique has recently been applied to patients with neuromuscular scoliosis [23,24,25], the aim of this study was to evaluate its suitability in women with Rett syndrome, focusing on radiological outcomes, weight gain, and complication rates.

## 2. Surgical Technique

A preoperative 1-month halo-gravity traction was used for stiff curves in 3 cases.

The patient was positioned on Jackson’s table with intra-operative traction and monitored by somatosensory potentials (SSEPs) and motor potentials (MEPs) [26,27]. The surgical approach consisted of two short longitudinal incisions, one located on the proximal thoracic spine and a second one at the lumbosacral region. A standard Wiltsee trans-muscular approach was used to reach the lumbosacral junction.

The standard bipolar construct extending from T1 to the pelvis was performed. An extension up to C7 could be considered in the absence of sufficient head control. The bipolar spinal fixation technique included proximal fixation by two pairs of double pedicle-supralaminar hook claws and distal fixation at the level of S1 using two ilio-sacral screws, inserted percutaneously using a dedicated guide (E.SPINE, TANIT, EUROS, La Ciotat, France) [28,29]. Two long pre-curved rods (titanium or chromium–cobalt—5.5 mm diameter) were fixed to the proximal hooks and tunnelled through the submuscular way towards the distal incision. The other two pre-bent short rods were fixed to the ilio-sacral screws by connectors. Then, a couple of rods were connected together by a closed side-to-side domino [22]. The reduction was obtained by applying concave distraction and in situ rod-bending maneuvers. The bipolar concept relies on a continuous tension between the proximal and the distal points of fixation obtained with rod lengthening on demand in the case of major residual pelvic obliquity or in the case of trunk imbalance on the frontal or sagittal plane [28]. The alternative (used in two of our patients, at the Risser stage 0) was the use of one self-expanding rod (NEMOST, EUROS, La Ciotat, France): the expansion can occur passively during the patient’s daily movements, during the bony growth of the spine, or actively during symmetric or asymmetric axial traction exercises of the trunk [23].

Postoperative care consisted of a short stay in the continuing care unit before being transferred to the orthopedic ward. The bipolar construct was strong enough not to require a postoperative brace. Additional surgery was performed if the curve worsened due to spinal or pelvic imbalance secondary to a growth spurt (rod-lengthening) or in the case of rod breakage.

## 3. Patients and Methods

### 3.1. Study Design and Data Collection

We reviewed 22 patients who underwent bipolar spine surgery for Rett syndrome in our department between 2015 and 2021. Ethical clearance was obtained through the European Union Drug Regulating Authorities Clinical Trials Database (ID-EUDRACT #201A01043-44). Informed consent for participation in the study was obtained from patients and their parents or a legal representative for the anonymized image reproduction.

We recorded the clinical (weight, comorbidities, ambulation; see Table 1) and radiographic data for each patient (Cobb angle, pelvic obliquity, thoracic kyphosis, and T1-S1 length; see Table 2 and Table 3) before surgery, immediately after surgery and at the last follow-up. We stated Risser of 4 or 5 as skeletal maturity.

We reviewed the following complications: respiratory failure and any other medical complications, mechanical complications (malposition of the ilio-sacral screw, migration of the hooks, or implant breakage), and infections.

### 3.2. Statistical Analysis

Continuous variables were described as the mean. Complications were described as n (%).

A Wilcoxon test was used to determine the weight change between pre and postoperative times and at follow-up. Changes in radiological parameter values from the baseline to the last follow-up were evaluated by applying the pairwise Student’s *t*-test. *p* values < 0.05 were considered significant.

## 4. Results

### 4.1. Demography

**Figure 1:**
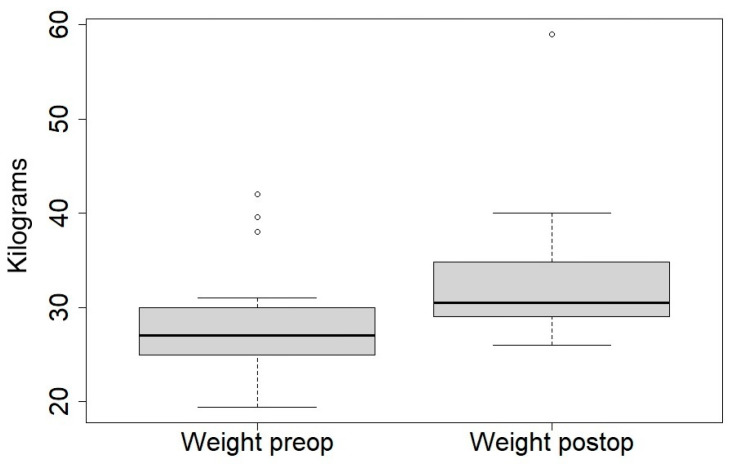
Boxplot of preoperative and postoperative weight.

We excluded 14 patients with a Rett-like syndrome (CKDL5, FOXG1, etc.). The mean age at initial surgery was 152 months (79–210). The mean follow-up was 56 months (24–99). Two patients were ambulatory. We recorded three deaths unrelated to surgery and after a minimum follow-up of two years. The mean body weight was 28 kg preoperatively and 33 kg at the latest follow-up (Figure 1). The main population characteristics are shown in Table 1.

In Figure 1, the body weight differences before and after surgery are shown.

### 4.2. Radiological Outcomes

Table 2 shows the main radiological parameters. There was no correlation between age and the Cobb angle.

**Figure 2:**
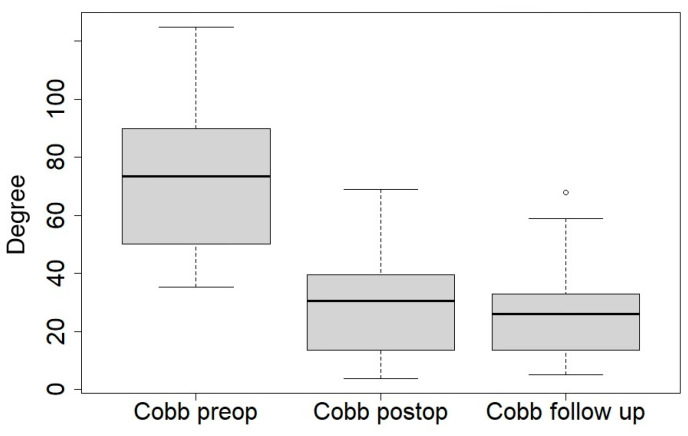
Boxplot of Cobb angle at preoperative time, postoperative time, and at last follow-up.

**Figure 3:**
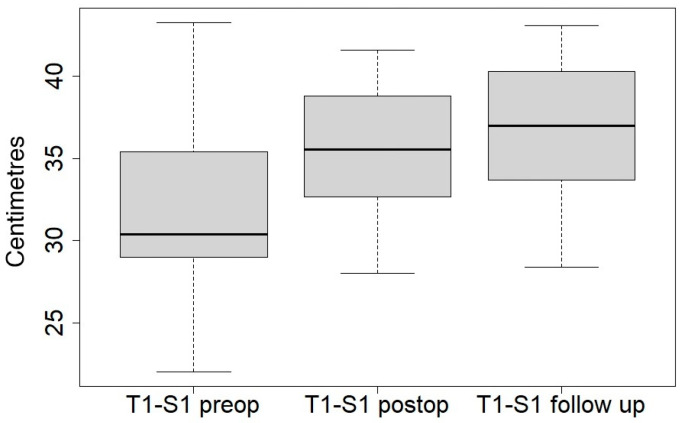
Boxplot of T1-S1 length at preoperative time, postoperative time, and at last follow-up.

Table 3 compares the main radiological changes between the preoperative, postoperative time, and last follow-up (Figure 2 and Figure 3).

For two young patients at Risser stage 0, we used the self-expanding NEMOST rods.

Thanks to a stable correction after initial surgery, 13 patients did not require rod lengthening. Nine patients required only one rod lengthening at a mean delay of 37 months (18–65) to achieve the further correction of spinal deformity and/or pelvic obliquity.

No screw loosening was recorded. We had two cases of osteolysis around ilio-sacral screws without clinical symptoms.

Skeletal maturity (Risser stage 4) was reached in 12 patients (55%) at the last follow-up.

The mean Cobb angle was 25.7° (5–68), and the mean pelvic obliquity was 9.4° (0–28.2) at the last follow-up. No patient required spinal fusion.

### 4.3. Complications

The global rate of immediate medical complications was 36%. The global rate of surgical complications was 31.8%; five infections were detected, with four deep infections treated by an unplanned return to theatre and one superficial infection treated by local care. Table 4 shows the details of the complications.

## 5. Discussion

This is a retrospective monocentric case review of Rett syndrome affecting scoliotic women treated with a minimally invasive bipolar technique construct.

This is the first study to evaluate the fusionless technique in Rett syndrome-induced scoliosis.

The patient selection was based on a single gene mutation (MCEP2) [3]. It is known that Rett syndrome affects patients who gradually lose weight as the scoliosis progresses during the late motor deterioration phase [18].

The surgery for scoliosis was performed in a national referral centre between 2015 and 2021 with a minimum of 2 years follow-up after initial surgery. We treated and analyzed twenty-two patients.

The aim of this study was to assess the efficiency of bipolar technique treatment in patients with severe and progressive scoliosis [11,12,13,18] requiring surgical treatment. The current treatment of Rett syndrome scoliosis is not univocal and is frequently based on PSF [14,30] and TGR. The goal of the treatment we performed was to let the spine and the chest grow [31]. Untreated scoliosis leads to additional thoracic insufficiency and cardiopulmonary disease. PSF prevents the lungs from growing (with limited alveolar growth potential and ventilatory defects) [32].

Posterior vertebral arthrodesis remains the most frequently used procedure in RS scoliosis, but it brings a large number of complications [30,31] and does not allow secondary curve correction and spine/pelvic balance [33]. The scoliotic curves in these young patients are flexible and can be corrected with posterior-only instrumentation.

The bipolar construct allows additional correction to be performed by symmetrical or asymmetrical rod lengthening, without fusion or fixation at the apex of the deformity, avoiding early auto-fusion and balancing the residual deformity and the pelvic obliquity. There are no other studies that explore the efficiency of the fusionless technique, specifically on Rett syndrome scoliosis.

Despite the poor bone quality of these patients, there was no screw pullout or construct failure throughout the follow-up. The minimally invasive bipolar technique allows spinal growth. We recorded a tangible trunk and pulmonary growth: pulmonary and gastroenterological complications decreased. Indeed, patients experienced a gain in weight (from 28 kg to 33 kg) (*p* < 0.01), similar to other studies from the literature [34].

**Figure 4:**
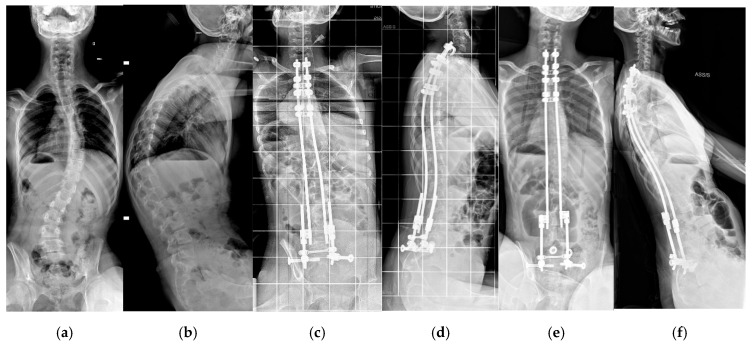
A 12-year-old girl operated on with the self-expanding rods: (**a**,**b**) preoperative AP and lateral X-rays; (**c**,**d**) in postoperative X-rays (**e**,**f**) at 3 years follow-up, we observed spontaneous and complete rod expansion thanks to bone growth.

**Figure 5:**
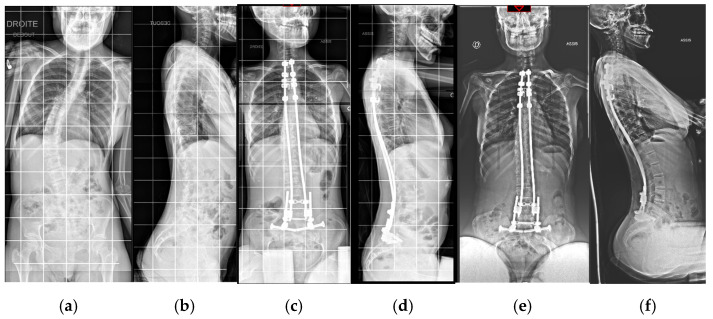
A 17-year-old girl operated on with self-expanding rods: (**a**,**b**) preoperative AP and lateral X-rays; (**c**,**d**) postoperative X-rays, (**e**,**f**) at 8 years follow-up.

In addition, bipolar fixation improves the quality of life of the patient as it improves comfort in the sitting position and avoids the need to use a brace, which is generally not well tolerated by patients. The early pulmonary complication rate was considerably lower (22%) compared to arthrodesis (48–63%) [30,31]. There was a significant number of perioperative infections compared to the literature on neuromuscular scoliosis [28,29,35,36], but we know that the incidence is higher in this specific disease [37]. A self-expanding device ideally avoids iterative surgical rod lengthening and the anesthesiologic-related risks (Figure 4a–f and Figure 5a–f). Thus, we were able to reduce iterative anesthetic procedures, decreasing wound-related complications and hospital admissions for children and caregivers.

In this study, there was no correlation between age and Cobb angle.

The mean Cobb angle improved from 74.4° preoperatively to 28.9° postoperatively (*p* < 0.001), with a gain of 61,2% and 25.7° at the last follow-up, corresponding to a gain of 65.5%.

Coronal balance improved during treatment.

The mean spinal length (T1-S1) improved from 31.8 to 35.7 cm after initial surgery (gain of 14.1%) and continued to improve to 36.4 cm at the last follow-up (gain of 18.2%), proving that this technique is effective at maintaining a good chest growth.

The use of a self-expanding device was an additional gain in the bipolar technique.

Prolonged instrumentation induces progressive stiffness in the spine over time, presenting the possibility of not converting the fusionless bipolar construct in final arthrodesis as the correction is maintained, with a substantial progressive and delayed auto-fusion of the spinal segments spanned. We had no rod breakage in this series.

Now, with more than ten years of follow-up using the minimally invasive bipolar fusionless technique with a stable result at the last follow-up, we can say that this technique is a real alternative to arthrodesis. We recommend performing it around the age of 10 years.

The law of diminishing return [38], due to early auto-fusion, was avoided in the present study, but late spinal stiffening due to progressive ankylosis in instrumented spines was observed [39,40].

In other published studies using shear wave and computed tomography analysis [39,40], it was shown that after five years from the last surgery, progressive ankylosis appeared, notably due to the reduction in disc width and articular process degeneration: the presence of metal rods is an auto-fusion and osteo-induction factor.

Thanks to this delayed spinal ankylosis, none of our patients required spinal fusion at skeletal maturity (55% of skeletal maturity).

Additional surgery was performed to improve spinal or pelvic residual imbalance, and in case of curve progression, this was due to growth spurts and infections.

Even with the relative rarity of the disease, the reduced number of patients marks the limitation of this study.

## 6. Conclusions

The results of this study indicate that the minimally invasive bipolar technique is a useful tool in the surgical management of severe spinal deformities in Rett syndrome.

This technique preserves spinal and thoracic growth and brings real benefits to patients, such as the improvement of general status, respiratory and digestive conditions, and the avoidance of final fusion thanks to the stable results of the correction over time.

The use of recent self-expanding rods avoids repetitive surgeries, reducing the risks of complications, especially septic ones, and thereby, the overall cost of treatment.

## Figures and Tables

**Table 1:** Main features of the 22 patients treated with fusionless surgery in Rett syndrome.

Unrelated death	3
Age at surgery, months, mean (range)	152 (79–210)
Body weight, kg	
Preop	28 (19–42)
Postop	33 (26–59)
Comorbidities	
Pulmonary issues	5/22
Gastro-intestinal issues	9/22
Orthopedic problems	5/22
Osteoporosis	11/22
Halo	3/22
Ambulatory	2/22

**Table 2:** Preoperative radiological parameters.

Cobb angle, °, mean (range)	74.4 (35.2–125)
Pelvic obliquity, °, mean (range)	30 (0–100)
Hyperkyphosis T4–T12 > 50°, °, mean (range)	66 (60–79)
Patients at skeletal maturity (Risser > 4), n, %	5 (23%)
C-shaped spine	14/22

**Table 3:** Comparison of radiographic data.

	Preoperative	Postoperative	*p*-Value (Preop/Postop)	Last Follow-Up	*p*-Value Postop/Follow-Up
Cobb angle, °, mean, range	74.4 (35.2–125)	28.9 (3.6–68.8)	<0.01	25.7 (5–68)	<0.05
T1-S1 length, mean, cm	31.8 (22–43.3)	35.7 (28–41.6)	<0.01	36.7 (28.4–43.1)	>0.1
Pelvic obliquity	29.5 (0–100)	9.7 (0–28.9)	<0.01	9.4 (0–28.2)	>0.01

**Table 4:** Complications.

Complications	Total Number (%)
Immediate medical	8 (36.3)
Respiratory c.	5 (22.7)
Non-invasive ventilation (NIV)	3 (13.6)
Pneumonia	2 (9.1)
Urinary infections	3 (13.6)
Surgical complications	7 (31.8)
Mechanical complications	2 (9.1)
Proximal hook migration	0
Ilio-sacral screw osteolysis	2 (9.1)
Rod breakage	0
Need for unplanned surgery	1 (4.5)
Infectious complications	5 (22.7)
Return to operating theatre	4 (18.2)

## Data Availability

The data are unavailable due to privacy or ethical restrictions.
